# The Regulatory Functions of lncRNAs on Angiogenesis Following Ischemic Stroke

**DOI:** 10.3389/fnmol.2020.613976

**Published:** 2021-02-04

**Authors:** Li Gan, Shengtao Liao, Yu Xing, Shixiong Deng

**Affiliations:** ^1^Laboratory of Forensic and Biomedical Information, Chongqing Medical University, Chongqing, China; ^2^Department of Gastroenterology, Second Affiliated Hospital of Chongqing Medical University, Chongqing, China

**Keywords:** long non-coding RNAs, ischemic stroke, angiogenesis, endothelial cells, therapeutic targets

## Abstract

Ischemic stroke is one of the leading causes of global mortality and disability. It is a multi-factorial disease involving multiple factors, and gene dysregulation is considered as the major molecular mechanisms underlying disease progression. Angiogenesis can promote collateral circulation, which helps the restoration of blood supply in the ischemic area and reduces ischemic necrosis following ischemic injury. Aberrant expression of long non-coding RNAs (lncRNAs) in ischemic stroke is associated with various biological functions of endothelial cells and serves essential roles on the angiogenesis of ischemic stroke. The key roles of lncRNAs on angiogenesis suggest their potential as novel therapeutic targets for future diagnosis and treatment. This review elucidates the detailed regulatory functions of lncRNAs on angiogenesis following ischemic stroke through numerous mechanisms, such as interaction with target microRNAs, downstream signaling pathways and target molecules.

## Introduction

Stroke is a type of acute cerebral vascular disease with high disability and mortality rate. It is the second leading cause of death and the third leading cause of disability worldwide (Mozaffarian et al., 2016; Feigin et al., 2017). The age-adjusted stroke mortality rate was 35/100,000, and there were 16.9 million cases of incident first stroke in 2010. Recently, the incidence of stroke increases rapidly and it is the leading cause of death in China, as over two million new cases were diagnosed in 2017 (Feigin et al., 2014; Wu S. et al., 2019). Ischemic stroke accounts for ~80–85% of all acute strokes, and its prevalence remarkably increases with advancing age (Randolph, 2016). Ischemic stroke is caused by cerebral artery occlusion, which reduces cerebral blood flow and causes neuronal cell death and brain dysfunction. The quality of life of patients can be severely affected by brain injury induced neurological deficits, leading to growing financial burden of health care.

It is well-established that early recanalization of blood vessels contributes to the restoration of blood supply in ischemic zone, and reduction of cerebral infarction area is the most effective treatment for acute ischemic brain injury. Angiogenesis following ischemic brain injury serves essential roles in clinical practice. Recent studies have indicated that enhancement of angiogenesis is a promising strategy for the treatment of ischemic stroke (Sun et al., 2020). Further understanding the detailed molecular mechanisms of post-ischemic stroke angiogenesis will facilitate the development of therapeutics.

Long non-coding RNAs (lncRNAs) are endogenous non-coding RNAs, which are >200 nucleotides in length but lack of open-reading frame. LncRNAs have been identified as key factors in regulating the expression and function of protein-coding genes through numerous mechanisms. LncRNAs with complex secondary and tertiary structure serve essential roles on epigenetics, transcription, and post-transcriptional modification via binding with target DNA, RNA or protein (Mousavi et al., 2013; Toiyama et al., 2014). LncRNAs are key regulators in biological processes such as cell proliferation, differentiation, apoptosis, autophagy, and angiogenesis, and they are involved in the pathogenesis of various types of diseases including tumors (Takahashi et al., 2020; Zhang Y. et al., 2020), cardiovascular disorders (Mao et al., 2019; Bian et al., 2020), and nervous system diseases (Liang et al., 2020). LncRNAs are considered as novel regulators in the pathogenesis of ischemic stroke. A recent study has suggested that lncRNAs are involved in the regulation of post-ischemic stroke gene expression (Yan Y. et al., 2020). Clinical research has also revealed that dysregulation of lncRNAs could be an essential mechanism in ischemic stroke (Huang J. et al., 2019). Therefore, lncRNAs could be novel therapeutic candidates in future targeted therapy.

However, the detailed roles and underlying mechanisms of lncRNAs on the pathogenesis of ischemic stroke remain largely unknown. Therefore, in order to explore putative therapeutic targets, this review highlights the importance of specific lncRNAs in angiogenesis following ischemic stroke.

## Biological Functions of LncRNAs

LncRNAs regulate gene expression through affecting epigenetics, transcription and translation. They serve essential physiological and pathological roles and are involved in numerous signaling pathways during the development of various diseases. LncRNAs are also considered as potential biomarkers. A previous study has indicated that H19 can target ACP5 protein directly and is a putative risk factor of ischemic stroke (Huang Y. et al., 2019). According to the subcellular distribution of lncRNAs, they are localized within the nucleus or cytoplasm, and previous studies have revealed their regulatory functions on gene expression at both transcriptional and post-transcriptional levels.

LncRNAs are involved in the regulation at the transcriptional level through acting as chromatin remodelers and transcriptional coactivators. Nuclear lncRNAs can regulate the functions of chromosomes by recruiting chromatin modifying complexes to specific sites on chromosomes, which consequently affects histone methylation and deacetylation; alternatively, lncRNAs can bind to regulatory transcription factors and modulate the transcription of target genes (Zhang C. L. et al., 2017; Lei et al., 2019; Zhang L. et al., 2019). At post-transcriptional level, cytoplasmic lncRNAs can regulate the expression of target gene indirectly through the downstream miRNAs (Zhuang et al., 2019). LncRNAs can also affect pre-mRNA splicing, mRNA translation and localization. For instance, some lncRNAs contain the complementary binding sites of target miRNAs and function as competing endogenous RNA (ceRNA) through binding certain miRNA competitively, which results in the upregulation of target mRNA (Cao et al., 2020). In addition, due to the existence of lncRNAs base-pairing with pre-mRNAs, the splicing of pre-mRNAs could be prevented, which subsequently affects the transcription of target genes (Szcześniak and Makałowska, 2016). Furthermore, certain lncRNAs are involved in the pathophysiology of diseases through binding to mRNA directly, which influences the degradation and translation of mRNAs (Wu H. et al., 2019).

## Regulatory Mechanisms of LncRNAs in Ischemic Stroke

LncRNAs are a group of non-coding RNAs involved in numerous biological processes and they are also considered as regulatory factors in ischemic stroke. In the human genome, ~40% of differentially expressed lncRNAs were detected in the brain and localized in specific cell types and subcellular structures in neural regions, which serve essential roles during brain injury (Derrien et al., 2012; Wang et al., 2017b). A recent study revealed significant alterations of lncRNAs in the brain after cerebral ischemia, suggesting the important physiological and pathological roles of lncRNAs on initiating endothelial responses to ischemic stimuli at transcriptional and translational level. Differentially expressed lncRNAs were detected in blood samples from patients with acute ischemic stroke, rat models of middle cerebral artery occlusion (MCAO), and oxygen/glucose deprivation (OGD) cells (Guo et al., 2018; Barangi et al., 2019; Zhu et al., 2019). The abovementioned lncRNAs are involved in various biological processes of ischemic stroke including cell proliferation, apoptosis (Zhang L. et al., 2020), oxidative stress, inflammation (Zhang and Zhang, 2020) and autophagy (Luo et al., 2020). Previous studies suggested that lncRNAs exert various regulatory functions after stroke. For example, they act as miRNA sponges competing with mRNA for miRNA binding. Moreover, lncRNAs could also regulate the expression of target genes directly through numerous signaling pathways.

### Regulation of Neurogenesis

Previous studies have revealed that altered levels of lncRNAs were associated with neurogenesis after stroke. A recent study indicated that N1LR inhibited neuronal apoptosis after ischemic stroke through inactivating p53 (Wu et al., 2017). Similarly, MEG3 interacted with p53 to mediate neuronal death (Yan et al., 2016). Furthermore, knockdown of MEG3 could suppress neuronal death by targeting the miR-21/PDCD4 signaling pathway (Yan et al., 2017). MEG3 induced autophagy and neuronal cell death via the miR-378/GRB2 axis, which subsequently suppressed the activation of Akt/mTOR signaling (Luo et al., 2020).

A previous study also indicated that MALAT1 promoted neuronal cell death and suppressed autophagy through targeting miR-30a in ischemic stroke (Guo et al., 2017). In consistence with these findings, H19 induced neuronal death by activating autophagy in ischemic stroke (Wang et al., 2017a). In addition, GAS5 inhibited cell death and suppressed neuronal survival through targeting the miR-137/Notch1 signaling pathway (Chen et al., 2018). Furthermore, SNHG14 promoted neuronal apoptosis through microglia activation via regulating the miR-145-5p/PLA2G4A axis in cerebral infarction (Qi et al., 2017).

### Regulation of Inflammation

Proinflammatory cytokines were released after ischemic brain injury, consequently activating NF-κB pathway that is involved in inflammatory responses. Nespas inhibited proinflammatory cytokine production and suppressed NF-κB signaling after ischemic stroke through TAK1 activation (Deng Y. et al., 2019). Gm4419 promoted neuroinflammation by inducing IκB phosphorylation and NF-κB signaling activation after ischemic stroke, and the levels of tumor necrosis factor-α (TNF-α), interleukin-1β (IL-1β) and IL-6 were increased (Wen et al., 2017). A previous study also indicated that H19 promoted neuroinflammation by M1 microglial polarization, which lead to increased production of TNF-α and IL-1β in ischemic stroke (Wang et al., 2017b).

### Regulation of Angiogenesis

Previous studies have suggested that lncRNAs serve essential roles on the regulation of endothelial cell survival, vascular integrity and angiogenesis in stroke. In this review, the mechanisms underlying lncRNA-modulated angiogenesis are summerized.

**Figure 1:**
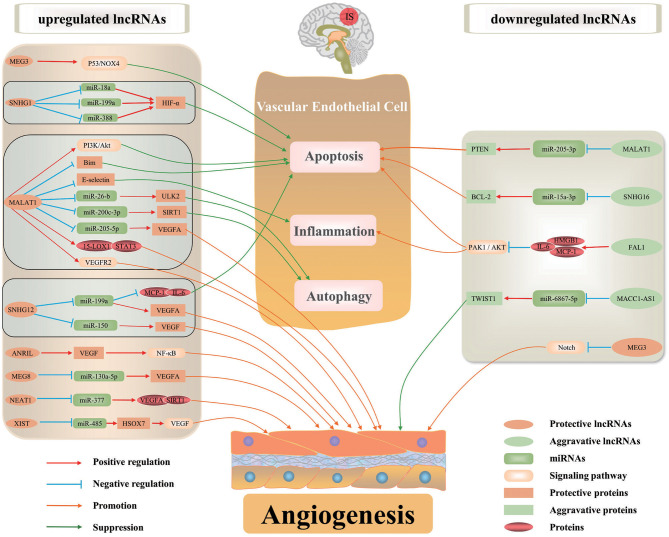
The regulatory mechanisms of lncRNAs in ischemic stroke-induced angiogenesis. Some lncRNAs serve essential roles on angiogenesis after ischemic stroke by regulating inflammation, autophagy and apoptosis of BMECs, or directly regulating angiogenesis-associated proteins/signaling pathways to maintain vascular function and integrity. Certain lncRNA may regulate the expression of multiple target genes in various pathways involved in angiogenesis.

Recent studies have revealed altered expression of lncRNAs in ischemic stroke (He et al., 2018; Ruan et al., 2020). Recently, lncRNA-mediated gene regulation has been intensively investigated. LncRNAs can regulate gene expression in ischemic stroke through affecting mRNA stability and epigenetic modifications. They function as decoys, scaffolds and enhancer RNAs of corresponding targets to regulate their functions. Furthermore, lncRNAs can interact with DNA and RNA by modulating pre-mRNA splicing or acting as miRNA sponge, resulting in impaired miRNA expression and functions. Numerous lncRNAs are associated with angiogenesis after ischemic stroke through affecting the transcription and translation (Figure 1, Table 1).

**Table 1:** The roles of lncRNAs on angiogenesis in ischemic stroke.

**lncRNA**	**Change in expression**	**Target miRNA**	**Target protein**	**Signaling pathway**	**Function**	**Reference(s)**
MEG3	Decreased			Notch	Promote angiogenesis	Liu et al., 2017
MEG3	Increased			P53/NOX4	Promote apoptosis; inhibit angiogenesis	Zhan et al., 2017
MEG3	—	miR-9			Suppress proliferation and angiogenesis	He et al., 2017
MEG8	Increased	miR-130a-5p	VEGF-A		Promote cell viability, migration, angiogenesis	Sui et al., 2020
MALAT1	—			CREB/PGC-1α/PPARγ	Protect cerebrovascular endothelial cells and BBB integrity	Ruan et al., 2019
MALAT1	Increased/decreased			PI3K/Akt	Inhibit apoptosis	Xin and Jiang, 2017; Wang G. et al., 2018
MALAT1	Increased			VEGFR2	Promote angiogenesis	Zhang X. et al., 2018
MALAT1	Increased			15-LOX1/STAT3	Promote proliferation, migration, angiogenesis	Wang C. et al., 2019
MALAT1	Increased			MDM2/P53	Promote apoptosis	Zhang T. et al., 2018
MALAT1	Increased		E-selectin		Reduce inflammatory	Zhang X. et al., 2017; Yang et al., 2018
MALAT1	Increased		Bim		Reduce apoptosis	Zhang X. et al., 2017; Yang et al., 2018
MALAT1	Increased	miR-126		PI3K/Akt	Inhibit proliferation and induce apoptosis	Zhang L. et al., 2020
MALAT1	Increased	miR-26b	ULK2		Activate autophagy; suppress apoptosis	Li Z. et al., 2017
MALAT1	Increased	miR-200c-3p	SIRT1		Activate autophagy; suppress apoptosis	Wang S. et al., 2019
MALAT1	Increased	miR-205-5p	VEGFA		Promote proliferation, angiogenesis	Gao C. et al., 2020
MALAT1	Decreased	miR-205-3p	PTEN		Promote apoptosis	Gao and Wang, 2020
SNHG1	Increased	miR-18a	HIF-1α	HIF-1α/VEGF	Inhibit apoptosis, promote BMEC survival	Zhang L. et al., 2018
SNHG1	Increased	miR-199a	HIF-1α, VEGFA		Promote angiogenesis, promote BMEC survival	Wang Z. et al., 2018
SNHG1	Increased	miR-338	HIF-1α	HIF-1α/VEGF-A	Inhibit apoptosis	Yang and Zi, 2019
SNHG12	Increased	miR-199a	MCP1, IL-6, VEGFA		Inhibit inflammatory and cell death; promote angiogenesis	Long et al., 2018
SNHG12	Increased	miR-150	VEGF		Promote proliferation, migration, angiogenesis	Zhao et al., 2018
SNHG16	Decreased	miR-15a-5p	BCL-2		Promote cell apoptosis	Teng et al., 2020
ANRIL	Increased		VEGF	NF-κB	Promote angiogenesis	Zhang B. et al., 2017
FAL1	Decreased		MCP1, IL-6, HMGB1	PAK1/AKT	Promote apoptosis and inflammatory; inhibit proliferation	Gao M. et al., 2020
XIST	Increased	miR-485	SOX7	VEGF	Promote proliferation, migration, angiogenesis	Hu et al., 2019
MACC1-AS1	Decreased	miR-6867-5p	TWIST1		Promote apoptosis and oxidative stress, inhibit angiogenesis	Yan G. et al., 2020
NEAT1	Increased	miR-377	VEGFA, SIRT1		Promote angiogenesis	Zhou et al., 2019
Rmst	Increased	miR-150			Promote apoptosis; inhibit proliferation, migration	Qiao et al., 2020
MIAT	Increased	miR-204-5p	HMGB1		Inhibit angiogenesis	Deng W. et al., 2020

## Brain Microvascular Endothelial Cells (BMECs)

BMECs are major structural and functional elements of brain microvasculature, which serve key roles on maintaining the integrity of the blood-brain barrier (BBB) and brain homeostasis by responding to the changes in cellular microenvironment dynamically. Ischemic stroke could cause structural and functional injuries to cerebrovascular endothelial cells, consequently increasing cerebrovascular permeability and damaging the BBB, which can result in neuroinflammation and neuronal damage. In addition, ischemic stroke results in BMECs injury and death. During cerebral ischemia, BMECs are stimulated by inflammatory mediators, which activates proinflammatory responses.

Angiogenesis after ischemic stroke promotes collateral circulation and restores blood supply in the ischemic area, subsequently reducing ischemic necrosis caused by ischemic injury and reversing the nerve damage (Li L. et al., 2017). LncRNAs were abundantly expressed in vascular endothelial cells and involved in endothelial biology and physiological functions.

Zhang et al. (2016) evaluated the expression levels of lncRNAs in primary BMECs after OGD using RNA-sequencing (RNA-seq) and differentially expressed lncRNAs were detected, including 147 up-regulated and 70 down-regulated lncRNAs. Among them, MALAT1 and SNHG12 are the most up-regulated lncRNAs. Highly conserved binding sites of transcription factors on the promoter region of lncRNAs reveals potential transcriptional regulation of lncRNAs, which could be associated with angiogenesis and serve essential roles on the response to ischemic stimuli; however, the detailed functions of lncRNAs in cerebral vascular endothelial biology is unclear.

A previous study indicated that cerebral vascular damage was aggravated in MALAT1 knock-out (KO) mice, where larger cerebral infarct volume and more severe neurological deficits were detected in response to ischemic insults (Zhang X. et al., 2017). Activation of MALAT1 was able to protect cerebral microvascular endothelial cells from ischemic injury (Yang et al., 2018). Additionally, SNHG12 was significantly up-regulated in microvascular endothelial cells under OGD/R conditions. Overexpression of SNHG12 inhibited brain microvascular endothelial cell death and inflammatory response, but promoted angiogenesis following OGD/R. Furthermore, SNHG12 suppressed brain microvascular endothelial cell injury by targeting miR-199a (Long et al., 2018). In addition, MACC1-AS1 was down-regulated in hypoxia-induced human brain microvascular endothelial cells (HBMECs). Overexpressed MACC1-AS1 reduced cell apoptosis and oxidative stress, while promoting cell proliferation, migration, and angiogenesis; meanwhile, cell permeability was decreased, which can affect cell barrier function. Moreover, MACC1-AS1 exert protective function of anti-apoptosis, pro-angiogenesis and anti-HBMECs injury via targeting the miR-6867-5p/TWIST1 signaling (Yan G. et al., 2020). Overexpression of FAL1 could reverse OGD/R-induced injury and cell death in brain microvascular endothelial cells through ameliorating oxidative stress, suppressing the secretion of pro-inflammatory cytokines and activating the PAK1/AKT signaling pathway (Gao M. et al., 2020).

## BMECs and Inflammation

Accumulating evidence revealed the importance of inflammation in the pathogenesis of stroke, which involves the networking of numerous inflammatory cells and cytokines. Multiple inflammatory cells and cytokines participate in various stages of ischemic stroke. Aberrantly expressed lncRNAs in ischemic stroke are associated with the production of inflammatory factors and activation of various signaling pathways. Cerebral endothelial cells are stimulated by inflammatory mediators produced by neuronal cells in ischemic brains, which triggers proinflammatory activation. Activated cerebral endothelium could release inflammatory factors such as IL-1β, IL-6, MCP-1 and induce the activation of NF-κB pathway, consequently leading to cerebral ischemic injury (Shekhar et al., 2018). Moreover, OGD/R could aggravate inflammation by enhancing the production of proinflammatory cytokines. Additionally, lncRNAs could regulate cerebrovascular function in ischemic stroke through targeting apoptotic factors.

MALAT1 could bind to IL-6, E-selectin and MCP-1, which prevent OGD/R-induced inflammatory response in ischemic stroke by reducing the production of proinflammatory cytokines (Zhang X. et al., 2017; Yang et al., 2018). For instance, SNHG12 was up-regulated under OGD/R conditions in microvascular endothelial cells of mouse brain, which decreased the mRNA and protein levels of proinflammatory cytokines E-selectin, MCP-1 and IL-6, subsequently inhibiting the inflammatory response. Furthermore, SNHG12 exerts anti-inflammatory function in brain microvasculature cells after OGD/R by reducing ischemic cerebral vascular injury and parenchymal damage (Long et al., 2018). In addition, FAL1 was significantly down-regulated in OGD/R-stimulated HBMECs, and overexpression of FAL1 ameliorated OGD/R-induced oxidative stress by suppressing the production of IL-6, MCP-1 and HMGB-1. FAL1 might serve essential roles on regulating OGD/R-induced inflammation in HBMECs (Gao M. et al., 2020). LncRNAs were involved in the inflammatory response in BMECs, which might affect brain endothelial cell damage through modulating the release of inflammatory factors in BMECs.

## BMECs and Autophagy

Autophagy is a lysosome-dependent homeostatic process. As an endogenous defense mechanism, it might also inhibit cell death. It has been well-established that lncRNAs are involved in the pathogenesis of ischemic stroke through regulating cell autophagy, which protects BMECs from ischemic injury. Autophagy can be activated by OGD/R as a defense response to protect BMECs against OGD-induced injury. Furthermore, lncRNAs are novel regulators involved in autophagy, and they can affect autophagy through downstream miRNAs; however, the underlying molecular mechanisms remain unclear.

Li Z. et al. (2017) revealed that MALAT1 was up-regulated in BMECs following OGD/R, which activated autophagy and promoted cell survival through down-regulating miR-26b and up-regulating ULK2. Wang S. et al. (2019) suggested that MALAT1 stimulated autophagy activation in BMECs via the miR-200c-3p/SIRT1 signaling, subsequently down-regulating p62 and inhibiting cell death. At present, the molecular mechanisms of autophagy in ischemic stroke are still unclear. Therefore, the detailed mechanisms of lncRNA-modulated regulatory functions on autophagy in BMECs under OGD/R conditions require further investigation.

## BMECs and Apoptosis

Dysfunction in the blood-brain barrier caused by the apoptosis of microvascular endothelial cells is one of the key pathological features of ischemic stroke, which contributes to secondary damage and poor prognosis in patients. LncRNAs might be involved in the apoptosis of BMECs through targeting apoptotic factors or various signaling pathways associated with the development of stroke. A recent study indicated that OGD/R down-regulated FAL1 and further reduced the phosphorylation of PAK1 and AKT, consequently promoting OGD/R-induced cell death. FAL1 could serve essential roles on regulating OGD/R-induced endothelial damage in HBMECs (Gao M. et al., 2020). Xin and Jiang (2017) revealed that the levels of MALAT1 in cells under OGD-R conditions were significantly increased, but down-regulation of MALAT1 was detected as oxygen levels increased. Additionally, MALAT1 suppressed OGD/R-induced apoptosis and the activity of caspase-3 in HBMECs by activating the PI3K/Akt pathway. These findings suggested that MALAT1 could protect HBMECs against OGD/R-induced endothelial damage. Furthermore, a previous study indicated that the expression of MALAT1 was down-regulated in OGD-induced apoptosis and negatively correlated with the expression of miR-205-3p. Subsequently, down-regulation of MALAT1 induced a reduction in PTEN levels, which further promoted OGD-induced apoptosis (Gao and Wang, 2020).

However, a recent study also revealed OGD-induced up-regulation of MALAT1 and down-regulation of miR-126 in HBMECs. In addition, knockdown of MALAT1 activated the PI3K/Akt pathway, increased the expression of phosphorylated PI3K/Akt and inhibited cell apoptosis. Moreover, MALAT1 down-regulated the expression of miR-126, which subsequently suppressed the proliferation and enhanced the apoptosis through inactivating the PI3K/AKT pathway. Therefore, up-regulation of MALAT1 could promote the development of ischemic stroke (Zhang L. et al., 2020). Another study indicated that MALAT1 could induce cell apoptosis. MALAT1 activated p53 signaling through targeting MDM2, consequently promoting the progression of ischemic stroke (Zhang T. et al., 2018). Furthermore, a previous study suggested that the expression of SNHG1 was remarkably increased in BMECs under OGD conditions and reversely correlated with the expression of miR-18a. Additionally, SNHG1 acted as miR-18a sponge and regulated the expression of its endogenous target HIF-1α, which inhibited cell apoptosis and promoted the survival of BMECs via the HIF-1α/VEGF signaling (Zhang L. et al., 2018). Another study also revealed that the levels of SNHG1 and miR-338 were up-regulated in OGD-induced BMECs. Knockdown of SNHG1 reduced the expression of HIF-1α/VEGF-A and promote the apoptosis of BMECs through targeting miR-338. As a result, OGD-induced injury in BMECs was aggravated. Therefore, SNHG1 exerted protective function against OGD-induced damage in BMECs via sponging miR-338 and up-regulate the HIF-1α/VEGF-A signaling (Yang and Zi, 2019). Additionally, the expression of SNHG12 was significantly increased in BMECs under OGD conditions, which inhibited cell death through targeting miR-199a (Long et al., 2018). Furthermore, SNHG16 suppressed OGD-induced apoptosis in HBMECs via the miR-15a-5p/Bcl-2 axis (Teng et al., 2020). In addition, up-regulation of Rmst inhibited cell proliferation/migration and promoted the apoptosis of OGD-induced cells through targeting miR-150 (Qiao et al., 2020). Moreover, MIAT was overexpressed in OGD-treated BMECs, and it could affect the injury of BMECs after cerebral ischemia through targeting the miR-204-5p/HMGB1 signaling (Deng W. et al., 2020). Taken all together, previous studies revealed the involvement of numerous lncRNAs in the apoptosis of BMECs after ischemic stroke.

## BMECs and Angiogenesis

The key steps of angiogenesis include the proliferation and migration of blood vascular endothelial cells. The Notch and VEGF signaling pathways are involved in angiogenesis, which could regulate cell differentiation and migration, contributing to the formation of blood vessels. Ischemic stroke promoted the proliferation and migration of BMECs, subsequently activating angiogenesis. Angiogenesis is a complex process that involves the regulation of numerous genes. LncRNAs are novel key regulators associated with endothelial dysfunction after ischemic stroke. Thus, lncRNAs could be involved in angiogenesis of endothelial cells by maintaining vascular function and integrity through various pathways.

Michalik et al. (2014) revealed that silenced MALAT1 could suppress the proliferation of vascular endothelial cells, suggesting that MALAT1 was able to promote endothelial cell growth and might serve essential roles on protecting cerebral microvasculature against ischemic brain injury. Furthermore, lncRNAs regulated the expression of proximal protein, which are major angiogenic molecules, subsequently affecting the process of angiogenesis. In addition, MALAT1 could enhance angiogenesis by binding to VEGFR2 (Zhang X. et al., 2018). Wang C. et al. (2019) also indicated that OGD/R increased the expression levels of MALAT1, 15-LOX1, VEGF and pSTAT3. Knockdown of MALAT1 inhibited the proliferation and migration of BMECs, suggesting that MALAT1 could promote angiogenesis through activating the 15-LOX1/STAT3 axis.

Furthermore, lncRNAs might be involved in angiogenesis through numerous signaling pathways. A previous study indicated that HOTTIP promoted the growth of BMECs via activating the Wnt/β-catenin axis (Liao et al., 2018). Zhang B. et al. (2017) also suggested that the expression of ANRIL was significantly up-regulated in the infarcted tissues of MCAO rat model. In addition, overexpression of ANRIL enhanced the expression of VEGF and promoted the angiogenesis by activating the NF-κB signaling.

LncRNAs could also function as ceRNAs of miRNAs to regulate the expression of target mRNAs, further affecting the process of angiogenesis. For instance, MEG3 is a key regulator in the proliferation and angiogenesis of BMECs by acting as miR-9 sponge (He et al., 2017). Moreover, MEG8 was up-regulated in OGD-treated BMECs. Silenced MEG8 inhibited the viability, migration and angiogenesis of BMECs via targeting miR-130a-5p. These findings suggested that MEG8 could protect BMECs from OGD-induced injury by promoting the angiogenesis following ischemic stroke through the miR-130a-5p/VEGFA axis (Sui et al., 2020). In addition, the expression of NEAT1 was elevated in BMECs under OGD conditions, which promoted the angiogenesis in BMECs by targeting the miR-377/SIRT1/VEGFA axis (Zhou et al., 2019). Both SNGH1 and SNGH12 were remarkably up-regulated in BMECs under OGD/R conditions, which promoted the survival and migration of BMECs. SNGH1 and SNGH12 exert their functions through targeting miR-199a and up-regulating their downstream molecules VEGFA and FGFb (Long et al., 2018; Wang Z. et al., 2018). Similarly, another study has suggested that up-regulation of SNHG12 could facilitate the restoration of neurological function in the infarct boundary zone of MCAO mice via the miR-150/VEGF signaling. Infarct volume was reduced, and cell growth/angiogenesis was enhanced by up-regulated SNHG12, suggesting that SNHG12 could promote angiogenesis following ischemic stroke (Zhao et al., 2018). Furthermore, XIST induced cell proliferation, migration and angiogenesis in BMECs via the miR-485/SOX7 axis (Hu et al., 2019). Another study also revealed that MALAT1 expression was increased in OGD/R-induced HBMECs. Up-regulated MALAT1 promoted cell proliferation and served protective roles on angiogenesis by targeting the miR-205-5p/VEGFA signaling in HBMECs under OGD/R conditions (Gao C. et al., 2020).

In summary, these findings indicated that lncRNAs including MALAT1, HOTTIP, ANRIL, MEG3, MEG8, NEAT1, SNHG1, SNHG12 and XIST were involved in post-ischemic stroke angiogenesis. Abovementioned lncRNAs are key regulators in angiogenesis by acting as ceRNA or targeting downstream molecules/pathways directly.

## LncRNAs as Novel Diagnostic and Prognostics Biomarkers for Ischemic Stroke

Recently, numerous lncRNAs have been identified using genome-wide analyses, and they are involved in various physiological and pathological processes in ischemic stroke through the regulation at both transcriptional and post-transcriptional levels. Previous findings revealed alteration of lncRNAs expression profiles in peripheral blood samples from patients with ischemic stroke, suggesting that lncRNAs could be novel diagnostic and prognostic biomarkers for this disease. A total of 206 lncRNAs were differentially expressed in the specimens from ischemic stroke patients, including 70 up-regulated and 128 down-regulated lncRNAs (Deng et al., 2018). Xu et al. (2020) revealed differential expression of 1,096 lncRNAs in the exosomes isolated from blood samples of patients with ischemic stroke, including 307 up-regulated and 789 down-regulated lncRNAs. Moreover, lnc-CRKL-2, lnc-NTRK3-4, RPS6KA2-AS1 and lnc-CALM1-7 could be potential biomarkers for the early diagnosis of ischemic stroke. Furthermore, Guo et al. (2018) suggested that 1,250 lncRNAs were differentially expressed in the peripheral blood samples following ischemic stroke. Additionally, ENST00000568297, ENST00000568243 and NR_046084 were identified as novel diagnostic biomarkers for ischemic stroke.

Moreover, the levels of lncRNAs might be associated with the severity of stroke. A previous study was performed on the plasma samples from patients with ischemic stroke, and up-regulated levels of lncRNAs including H19, MIAT, ANRIL, MEG3 and NEAT1 were detected. Up-regulation of abovementioned lncRNAs were positively correlated with the National Institutes of Health Stroke Scale (NIHSS) scores and infarct volume, which could affect the progression of ischemic stroke. Furthermore, the levels of H19 and NEAT1 were associated with the production of inflammatory factors including TNF-α, IL-6, IL-8, and IL-22. In addition, the expression levels of MIAT, ANRIL and NEAT1 were related to the up-regulation of high-sensitivity C-reactive protein. Abovementioned lncRNAs were considered as novel biomarker for the diagnosis of ischemic stroke (Wang et al., 2017b; Zhu et al., 2018; Zhang K. et al., 2019). In contrast, previous findings also revealed that ZFAS1 was significantly down-regulated in patients with large-artery atherosclerosis (LAA) stroke compared to non-LAA stroke group (Wang J. et al., 2019).

Some lncRNAs are associated with the prognosis of patients with ischemic stroke. A previous study indicated that the expression of MIAT was correlated with the therapeutic outcome. MIAT could be an unfavorable indicator of functional recovery, as reduced MIAT levels were detected in patients with favorable outcome, and vice versa. More importantly, the risk of death was higher in high MIAT expression group compared with the patients with low MIAT expression, in whom better prognosis and survival rate were observed (Zhu et al., 2018). In consistence with these findings, Wang et al. (2020) also suggested that MEG3 expression was associated with poor prognosis in patients with ischemic stroke. The risk of death was increased in high MEG3 expression group compared with the control. Furthermore, the expression levels of NEAT1 in patients with recurrence were elevated compared with non-recurrence group. In addition, patients with high NEAT1 expression exhibited shorter RFS compared with low NEAT1 expression group (Li et al., 2020). These studies revealed the expression profiles of lncRNAs in patients with cerebral ischemia, suggesting that lncRNAs could be potential diagnostic and prognostic biomarkers for ischemic stroke.

Previous studies have indicated that microvessel density in the peri-infarct region was increased in patients with ischemic stroke. Angiogenesis was able to promote the recruitment of blood flow and metabolic nutrients into the infarct regions, subsequently facilitating functional recovery. Enhancement of angiogenesis is a promising therapeutic strategy for the treatment of ischemic stroke (Oshikawa et al., 2017; Zhang J. et al., 2019).

At present, the most effective treatment for ischemic stroke is vascular recanalization including interventional treatment and intravenous thrombolysis, which could restore the cerebral blood flow and reduce the infarction size. Until now, recombinant tissue plasminogen activator (rtPA) is the only FDA-approved drug for the treatment of ischemic stroke. However, the use of tPA is limited by its narrow therapeutic window and side effects including high risk of secondary bleeding. Patients with proximal occlusion in large arteries of the brain could be subjected to endovascular interventions. So far, no effective treatment is available for patients whose blood vessels cannot be recanalized at early stage. Therefore, new therapeutic strategies should be developed for patients with ischemic stroke, and enhancement of angiogenesis is a major target in the treatment of this disease.

RNA therapeutic strategies have been developed to intervene the angiogenesis after ischemic stroke. Therapeutic strategies by manipulation of lncRNAs have been evaluated in the treatment of cancer and ulcerative colitis (Velagapudi et al., 2017; Vautrin et al., 2019). RNA therapeutics mainly base on the inhibition or overexpression of certain RNAs. RNA knockdown technique was used to examine the functions of target RNAs. As lncRNAs are located in the nucleus and/or cytoplasm, antisense therapy and RNA interference (RNAi) are commonly used to induce the degradation of lncRNAs (Lennox and Behlke, 2016, 2020). MEG3 was upregulated in cerebral infarction area, and MEG3 knockdown vector was transfected into the striatum of ischemia/reperfusion rat, which reduced infarct size and promoted angiogenesis (Liu et al., 2017). Furthermore, treatment with nano polymer wrapped MEG3 shRNA conjugated with OX26 antibody was able to reduce the ischemic lesion volumes and enhance the angiogenesis in cerebral infarction area (Shen et al., 2018). MALAT1 was upregulated in mouse BMECs after OGD. Antisense LNA GapmeR was used to knockdown MALAT1, which increased cell death, aggravated inflammatory reaction and reduced vascular growth in BMECs (Michalik et al., 2014; Zhang X. et al., 2017). Chemical modifications of lncRNAs could also improve the therapeutic efficacy (Zhang H. et al., 2019). The effects of adeno associated virus (AAV), CRISPR/Cas9, gapmer antisense oligonucleotides (ASOs) remain to be further elucidated.

However, the translational value of most lncRNAs remains unclear. Firstly, in previous studies on the regulatory roles of lncRNAs in ischemic stroke, sample sizes are relatively small, and further large-scale cohort studies are required to confirm the existing findings. LncRNAs are not necessarily conserved in different species. Most studies on lncRNAs were performed on animals, but the effects of putative therapeutics on patients with ischemic stroke should be considered. Secondly, the detailed regulatory functions of lncRNAs should be identified. Better understanding of the mechanisms could improve drug development to reduce off-target effects. Furthermore, the complex nature of brain structure and function resulted in various regulatory mechanisms of lncRNAs in different types of brain cells involved in ischemic stroke, a better understanding of lncRNAs in specific cell types under normal and pathophysiological conditions could improve the specificity of lncRNA-targeted therapies. In addition, multiple lncRNAs are simultaneously involved in the regulation of angiogenesis after ischemic stroke, combinations of lncRNAs with different pathological targets could improve the therapeutic outcome of cerebral ischemia. The accuracy and stability of RNA-based therapeutics should be considered in clinical practice.

## Conclusion

Emerging evidence has suggested that lncRNAs serve essential roles in the pathogenesis of ischemic stroke. LncRNAs could regulate the biological bahaviors of BMVECs through their target genes and serve essential roles on cell apoptosis, autophagy, inflammation, and angiogenesis. Furthermore, lncRNAs were closely associated with the prognosis of patients with ischemic stroke, and they could facilitate functional recovery by activating angiogenesis. In addition, lncRNAs are involved in angiogenesis by acting as miRNAs sponges or targeting the downstream signaling pathways/proteins directly. Therefore, lncRNAs might be potential diagnostic and prognostic biomarkers for ischemic stroke. However, only a few lncRNAs involved in angiogenesis have been studied, including MEG3, MEG8, MALAT1, SNHG1, SNHG12, SNHG16, ANRIL, FAL1, XIST, MACC1-AS1, NEAT1, Rmst, and MIAT. Therefore, further investigation is required to elucidate the detailed functions of lncRNAs in ischemic stroke. A better understanding of the underlying mechanisms could provide novel insights on the development of therapeutic targets and diagnostic biomarkers for patients with ischemic stroke.

## Author Contributions

LG and SL wrote the manuscript. YX and SD edited the manuscript. All authors contributed to the article and approved the submitted version.

## Conflict of Interest

The authors declare that the research was conducted in the absence of any commercial or financial relationships that could be construed as a potential conflict of interest.
